# Sporadic Hemiplegic Migraine

**DOI:** 10.7759/cureus.38930

**Published:** 2023-05-12

**Authors:** Tina Kana, Saraf Mehjabeen, Nirav Patel, Ahmed Kawamj, Zaineb Shamim

**Affiliations:** 1 Internal Medicine, Touro College of Osteopathic Medicine, New York City, USA; 2 Internal Medicine, New York Medical College, Passaic, USA; 3 Internal Medicine, NYU Langone, New York City, USA

**Keywords:** motor weakness, dysphagia, hemiplegic migraine, hemi-paresthesia, migraine disorder

## Abstract

An uncommon presentation of a migraine headache is hemiplegic migraine, which can clinically imitate other conditions including transient ischemic attacks and stroke with unilateral muscle weakness or hemiplegia. We present a 46-year-old female patient who was admitted with symptoms of a unilateral occipital headache, dysphagia and left-sided motor weakness. Diffusion magnetic resonance imaging (MRI) and brain tomography results were normal. A diagnosis of sporadic hemiplegic migraine was made after extensive workup and managed conservatively with solumedrol. The patient was discharged on prednisone and tetrahydrozoline ophthalmic solution with a drastic improvement in symptoms. On a follow-up visit, there was a complete resolution of symptoms.

## Introduction

A rare variant of migraine with aura is sporadic hemiplegic migraine which presents with a migraine headache associated with motor weakness or hemiplegia in the absence of any family history [1]. Patients present with severe headache, photophobia, numbness, tingling, paresthesias, dysarthria and temporary muscle weakness which can last from minutes to days [2]. Migraine associated with hemiplegia is a rare presentation, and minimal data is available in terms of pathophysiology and treatment of sporadic hemiplegic migraine. Prompt diagnosis, however, can be extremely difficult given the multiple and rare forms of migraines. As a result, treatment is often frequently delayed at the expense of the patient.

## Case presentation

A 46-year-old female patient with no significant past medical history presented for evaluation of a left-sided headache which began four days before ED admission. She noted that there was a gradual onset of the headache, which was described as a throbbing sensation. The patient tried ibuprofen without alleviation of symptoms and denied trauma or any loss of consciousness. She developed right eye pressure and right-sided facial tingling in the morning which prompted her visit. The patient denied fevers, chest pain, extremity weakness, abnormal gait, recent travel or insect bites. On physical examination, the patient was able to speak in full sentences, but left facial asymmetry was present. Lyme serology was performed, and negative results were obtained. Brain tomography results were normal, and the patient was given ketorolac, methylprednisolone and metoclopramide. The patient’s symptoms improved over the next hour, and she was discharged with a diagnosis of Bell’s palsy. She was prescribed oral prednisone and acyclovir at the time of discharge.

**Figure 1:**
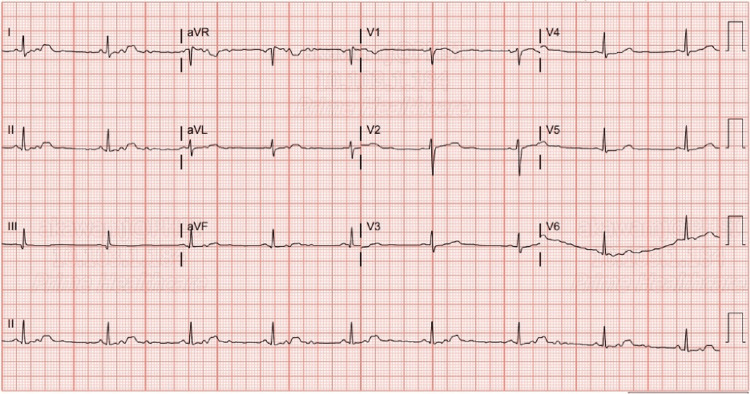
EKG: sinus rhythm with a rate of 52 beats per minute

**Figure 2:**
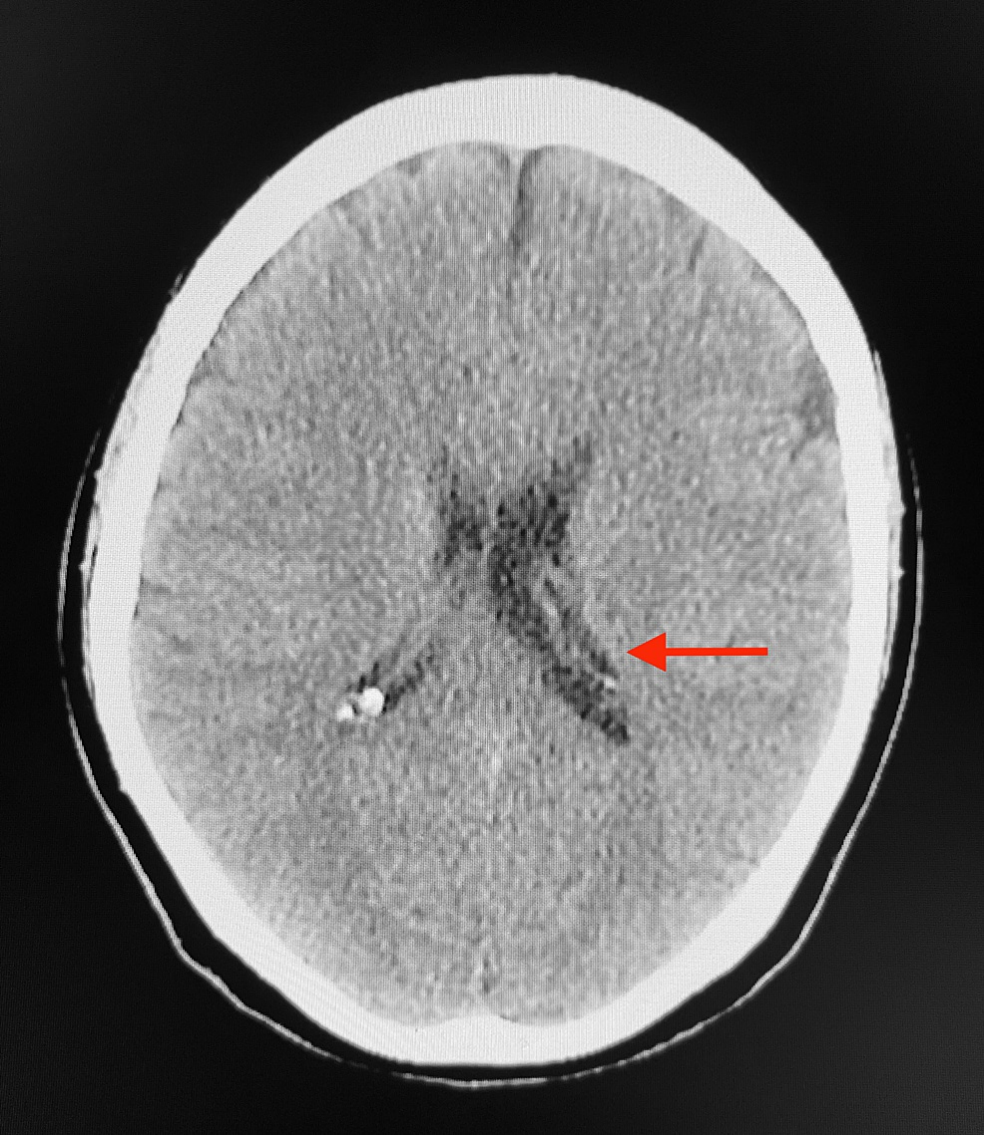
CT of the head without contrast: left lateral ventricle (red arrow) slightly larger than the right, a normal variant No significant intracranial findings.

**Figure 3:**
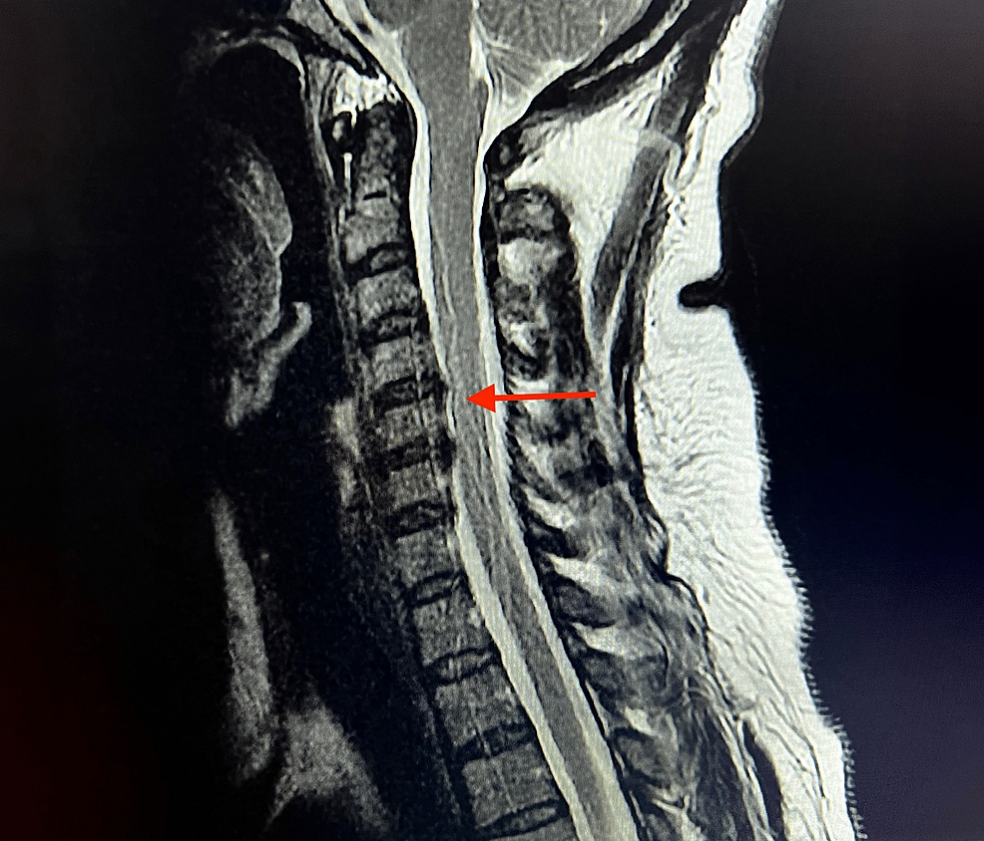
MRI of the cervical spine with and without contrast: mild spondylosis throughout the cervical spine and mild broad-based disc bulging (red arrow) at C5-C6 without any significant neural foraminal narrowing

Ten days later, the patient presented with a left-sided occipital headache and increasing weakness and numbness in her left upper and lower extremities, which had been ongoing for the last five days. She also reported left-sided facial pain, nausea, dysphagia, paresthesia and blurry vision in her left eye. On physical examination, the patient was afebrile, blood pressure was 122/75 mmHg, pulse was 59 beats per minute and respirations were 20 breaths per minute. Neurological examination revealed that the patient’s right upper and lower extremity strength was 5/5, and the left upper and lower extremity strength was 3/5. Romberg, shin-to-heel, pronator drift and Babinski testing were negative, and two-point discrimination was intact. Additionally, reflex testing was normal. An EKG revealed a normal sinus rhythm with a rate of 52 beats per minute (Figure 1). Complete blood count (CBC) and comprehensive metabolic profile (CMP) lab results were unremarkable, except for suboptimal B12 levels at 313.4 pg/mL (Table 1). A CT of the head without contrast revealed no significant intracranial findings with ventricles that were midline without evidence of hydrocephalus or shift. The left lateral ventricle was slightly larger than the right, which was considered a normal variant (Figure 2). An MRI of the brain without contrast indicated no acute intracranial hemorrhage, acute infarction, extra-axial collections or mass effect, and no acute pathology of the brain was seen. An MRI of the cervical spine with and without contrast revealed mild spondylosis throughout the cervical spine and mild broad-based disc bulging at C5-C6 without any significant neural foraminal narrowing (Figure 3).

**Table 1:** Select lab values BUN: blood urea nitrogen, AST: aspartate aminotransferase, ALT: alanine transaminase, GFR: glomerular filtration rate, TSH: thyroid-stimulating hormone, PT: prothrombin time, INR: international normalized ratio.

Test	Value	Reference Range
WBC	6.9 ×10^3^/µL	3.5-10.5 ×10^3^/µL
RBC	4.45 ×10^6^/µL	3.9-5.03 ×10^6^/µL
Hemoglobin	13.9 g/dL	12.0-15.5 g/dL
Hematocrit	40.3%	34.9%-44.5%
Sodium	139 mmol/L	136-145 mmol/L
Potassium	4.5 mmol/L	3.5-5.3 mmol/L
BUN	9.5 mg/dL	6.0-24.0 mg/dL
Creatine	0.6 mg/dL	0.5-1.0 mg/dL
Glucose	88 mg/dL	70-140 mg/dL
AST	12 U/L	10-36 U/L
ALT	18 U/L	6-29 U/L
Calcium	8.8 mg/dL	8.6-10.4 mg/dL
GFR	>60 mL/min/1.73 m^2^	>60 mL/min/1.73 m^2^
Vitamin B-12	313.4 pg/mL	200-900 pg/mL
TSH	0.787 µIU/mL	0.5-5.0 µIU/mL
PT	13.3 seconds	11.0-13.5 seconds
INR	0.99	<1.1
Lyme serology	Negative	Negative
HIV serology	Negative	Negative

The patient was started on IV methylprednisolone 125 mg BID and B12 1,000 mcg IM after consulting with neurology. Butalbital/acetaminophen/caffeine was administered throughout the hospital stay for symptomatic relief of migraine symptoms. The patient reported significant improvement in the migraine symptoms over the next three days and noted a slight improvement in hemiparesis. She was discharged with prednisone and tetrahydrozoline drops. On a two-week follow-up visit, the patient presented with full resolution of all symptoms.

## Discussion

According to the World Health Organization, 50%-75% of adults aged 18-65 years worldwide have experienced a headache within the last year. Up to 30% of those individuals reported having a migraine [3]. Migraines are characterized by paroxysmal headache associated with aura or without aura. Factors of pathogenesis are associated with cortical spreading depression, which activates the trigeminal nucleus caudalis, and is thought to originate in the occipital region and spread anteriorly. [4] It has been proposed that vasogenic leakage from leptomeningeal vessels stimulates the trigeminovascular system and contributes to the auras experienced in patients with hemiplegic migraines [5].

A rare aura migraine subtype is hemiplegic migraine, which was initially described in 1910 by Clarke [6]. The reported prevalence of hemiplegic migraine is 0.01% [7]. It is characterized by transient hemiplegia or hemiparesis that occurs during an attack of migraine headache. The hemiparesis may resolve before the headache or may persist for days to months. Hemiplegic migraine can be divided into two groups, familial and sporadic. Familial hemiplegic migraine demonstrates autosomal dominant inheritance with mutations in CACNA1A, ATP1A2 and SCN1A genes, as well as a positive family history of similar attacks [8]. Sporadic hemiplegic migraine should be high on the differential diagnosis, particularly in the absence of first- and second-degree relatives with similar disorders.

Our patient’s initial presentation was a severe throbbing unilateral occipital headache associated with left-sided facial pain, nausea, paresthesia and blurry vision of the left eye. Subsequently, over the next 48 hours, she developed additional symptoms with increasing left upper and lower extremity hemiparesis as well as dysphagia, which were initially attributed to a transient ischemic attack. However, the clinical presentation and normal findings on diffusion MR imaging and brain tomography indicated that the hemiparesis was not due to hemorrhage or ischemic stroke. Additional differential diagnoses for this patient included stroke, transient ischemic attack, brain tumor, Bell’s palsy, Lyme disease and meningitis. This imaging modality was negative for any masses, making brain tumor also unlikely. Bell’s palsy was a diagnosis that was indicated based on the symptoms. However, this condition typically produces symptoms isolated to facial nerve palsy. The patient's presentation, in this case, was not isolated to the distribution of the facial nerve alone, indicating the presence of some other cause. In addition, the patient was previously treated with steroids based on the assumption of Bell’s palsy, and the symptoms of hemiplegia progressed despite treatment. Serological testing was performed to evaluate for Lyme disease, and the results were negative. In addition, there was no history of tick exposure, rash, arthralgias or other supportive symptoms of this. Meningitis was considered as a possible cause; however, laboratory testing did not reveal any signs of infection including leukocytosis or elevated inflammatory markers such as the erythrocyte sedimentation rate. In addition, there was no fever, nuchal rigidity, mental status changes or signs of sepsis including hypotension, tachycardia and dyspnea. Based on the history, lab work and imaging, it was determined that the patient’s symptoms were indicative of a migraine aura. According to the International Classification of Headache Disorders (ICHD-3), we believe that our patient fulfills the diagnostic criteria for hemiplegic migraine (Table 2). It is classified as a migraine with a prolonged aura of hemiplegia. Familial hemiplegic migraine does not fit our patient due to a lack of family history [9].

**Table 2:** Representation of diagnostic criteria for hemiplegic migraine

1. Presence of aura, which consists of:
A. Reversible motor weakness
B. Visual, sensitive and/or speech/language symptoms that are completely reversed
2. At least two of the following:
A. At least one symptom of aura develops gradually over greater than five minutes
B. Two or more symptoms appear successively
C. Each individual aura symptom lasts 5-60 minutes
D. At least one aura symptom is unilateral
E. At least one aura symptom is positive
F. The aura is accompanied, or followed within 60 minutes, by headache
3. No first- or second-degree family member has diagnostic criteria for hemiplegic migraine

Presently, there are no standardized treatment guidelines for hemiplegic migraine. Due to the rarity of the condition, treatment trials have not been conducted on large groups of patients. Treatments reported in the medical literature have been part of single case reports or on a small series of patients. Treatment options can be divided into acute treatment during an attack, where medications are administered to reduce the severity of symptoms, and preventative treatment to reduce the frequency of attacks.

During an attack, treatment options include non-steroidal anti-inflammatory drugs (NSAIDs) and non-narcotic pain relievers. However, no acute treatment has been shown to have proven efficacy to reduce the intensity and duration of the aura associated with hemiplegic migraine. However, ergot derivatives and triptans are contraindicated due to their potential vasoconstrictive effects, which could potentially lead to permanent damage including an increased risk of stroke [10]. The patient in our case did not want to be prescribed an NSAID, as she stated that she had tried over-the-counter NSAIDs which proved to be ineffective. Neurology was consulted, and the decision was made to prescribe prednisone. Although there is no evidence supporting the use of specific anti-migraine medications to reduce the frequency of attacks, beta-blockers, calcium channel blockers, tricyclic antidepressants and anticonvulsants can be administered. Long-acting verapamil or lamotrigine can be used for more frequent sporadic episodes rather than for the first episode in an acute setting.

## Conclusions

It is paramount that physicians are able to identify life-threatening and non-life-threatening causes of headaches. Patients admitted for migraine headaches typically present with throbbing headaches, nausea, vomiting, photophobia and phonophobia. However, physicians should include rare variants such as hemiplegic migraine in their differential diagnoses especially when headaches are associated with neurological symptoms that include motor weakness in the upper and lower extremities, dysarthria, dysphasia and Bell’s palsy symptomatology.
